# Improving Four-pre ability for flood in alpine regions

**DOI:** 10.1016/j.eehl.2025.100189

**Published:** 2025-10-01

**Authors:** Li Zhou, Haowen Li, Zhongshun Gu, Yinan Guo, Xiaopeng Wang, Biqiong Wu, Lingling Wu, Chun Zhou, Zhipan Niu

**Affiliations:** a Institute for Disaster Management and Reconstruction, Sichuan University-Hong Kong Polytechnic University, Chengdu 610207, China; b State Key Laboratory of Hydraulics and Mountain River Engineering, College of Water Resource & Hydropower, Sichuan University, Chengdu 610065, China; c Xizang Autonomous Region Meteorological Information and Network Centre, Lhasa 850001, China; d China Three Gorges University, Yichang 443002, China; e Hubei Key Laboratory of Intelligent Yangtze and Hydroelectric Science, China Yangtze Power Co., Ltd., Yichang 443000, China; f Sichuan Hydrological and Water Resources Survey Center, Chengdu 610036, China

**Alpine regions face severe flood management challenges due to complex terrain, climate extremes, and sparse data. This commentary critiques limitations in current hydrological practices supporting China's Four-pre strategy. Key barriers include poor data quality, model limitations, and fragmented systems. Thus, we recommend optimizing observation networks, using physics-informed neural networks, and establishing unified data platforms. This integrated approach will enhance flood modeling reliability in data-scarce regions, providing actionable insights for adaptive flood governance in high-risk mountainous regions.**

Mountains in alpine regions, including the Himalayas, Andes, Rockies, and Alps [1], are vital for global ecological security and water resource management. Characterized by steep topography, pronounced elevation gradients, and highly heterogeneous climatic regimes, these regions host a rich diversity of ecosystems along with abundant glacier and snowpack resources. They constitute the headwaters of many of the world's major rivers, including the Yangtze, Ganges, Mississippi, and Danube, and exert critical influence on regional water budgets, hydrological dynamics, and the development of extreme flood events [2]. However, precipitation observations in such regions still suffer from significant deficiencies in spatial and temporal resolution, accuracy, and continuity, which limit the precision of hydrological modeling and the effectiveness of disaster response [3], thus becoming a key bottleneck in the implementation of the Chinese Ministry of Water Resources' Four-pre strategy: forecasting, early warning, simulation and contingency planning. These limitations manifest in several key aspects:

1) **Observations and insufficient data infrastructure**

Due to limited accessibility, high maintenance costs, and extreme environmental conditions, alpine rain gauge networks remain sparse and unevenly distributed, limiting their capacity to capture the spatial heterogeneity of precipitation, especially at meso-to small scales. Although satellite-based precipitation products offer wide spatial coverage, their accuracy declines sharply over high elevations, complex terrain, and snow-covered surfaces, often exhibiting systematic biases. Ground-based weather radars face similar challenges: topographic shielding frequently leads to under-detection or “missed” precipitation events, further weakening regional monitoring capacity. This persistent lack of reliable observations has not been fundamentally improved over time, resulting in substantial uncertainties in model inputs. Since precipitation forecasting models critically rely on accurate input data, the insufficiency and biases of observational datasets directly undermine the reliability of subsequent modeling, constituting a fundamental barrier to enhancing the predictive accuracy and operational efficacy of the Four-pre flood management system.

2) **Complex hydrological processes and modeling limitations**

Steep topography and pronounced vertical climatic gradients in alpine regions give rise to meteorological and hydrological processes that are highly nonlinear, spatially heterogeneous, and temporally dynamic. The coupled interactions among precipitation, snowmelt, and freeze-thaw dynamics further complicate flood generation mechanisms, making them difficult to anticipate. Even widely used process-based hydrological models remain highly sensitive to the quality of their input data. When the observational gaps and biases discussed earlier are incorporated into model forcing, errors rapidly accumulate, leading to a marked decline in simulation reliability and limiting the ability to capture localized, fast-responding, and small-to meso-scale extreme hydrological events.

3) **Fragmented data ecosystems and limited model adaptability**

Beyond the constraints of the natural environment, flood forecasting in alpine cold regions is further hampered by institutional and technical barriers. Observation data are managed under fragmented systems, with substantial discrepancies in governance, format standards, and sharing protocols across agencies and regions, creating entrenched “information silos”. Precipitation observations from meteorological bureaus, hydrological monitoring from water agencies, and station networks established by research institutions are often operated in isolation, lacking unified norms and interfaces that would enable integration and exchange.

Data fragmentation should not be regarded merely as an external obstacle comparable to environmental or technical challenges; instead, it functions as a systemic driver that reinforces and amplifies both. On one hand, it prevents the consolidation and sharing of already limited observation resources, perpetuating the sparsity and biases of existing datasets. On the other hand, it undermines the data support essential for precipitation-flood forecasting models, exacerbating their uncertainties and limitations. Under such structural constraints, even emerging data-driven approaches remain fundamentally limited by observation gaps and model complexity. In other words, fragmentation not only magnifies the core challenges of the “Four-pre” framework but also obstructs potential pathways toward their resolution.

To systematically enhance flood forecasting, early warning, simulation and contingency planning in alpine regions, we recommend a three-tiered integrated framework, as shown in Fig. 1, comprising an infrastructure foundation, a modeling and simulation core, and a governance and integration layer. Each layer supports and reinforces the others, forming a coherent strategy for adaptive flood management under complex alpine conditions:

1) **Optimizing observation networks and data infrastructure**

**Fig. 1:**
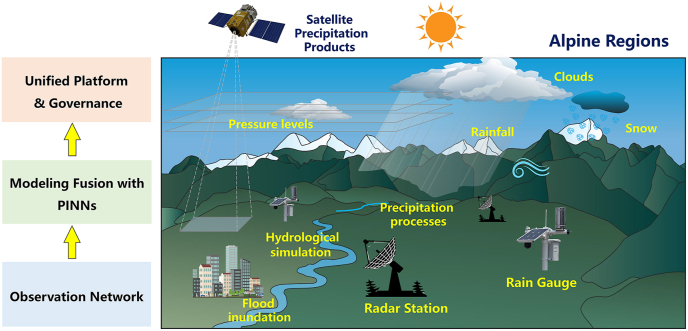
Systematic approach to alpine flood management.

Monitoring capacity fundamentally constrains the accuracy and scalability of hydrological modeling. In alpine regions, the substantial costs associated with the construction and maintenance of rain gauges and hydrological stations hinder the deployment of dense, regionally representative networks. The integration and cross-validation of multi-source precipitation products remain limited. We recommend that in regions where observational density is relatively higher, cross-validation and bias correction be used to evaluate the performance of alternative deployment strategies under complex terrain and climatic conditions. Insights from such evaluations can inform the design of optimal spatial configurations that not only maximize information value but also provide transferable references for sparsely monitored areas. This approach enables the establishment of structurally robust and spatially efficient monitoring systems under resource constraints and extreme environments, thereby providing a stronger foundation for data fusion and mountain flood modeling. Crucially, calibration-based network optimization also creates a spatial framework inherently compatible with multi-source datasets, minimizing the risk of new information silos and laying both institutional and technical foundations for cross-agency data sharing and model validation.

2) **Physics-informed neural networks for data fusion modeling**

Flood simulation in alpine regions faces dual challenges: sparse observations and inadequate physical consistency. Pure data-driven models often violate fundamental hydrological laws, such as mass conservation, while conventional physically based models struggle to adapt to complex topography and extreme events. We recommend advancing physics-informed neural networks (PINNs) [4], which integrate domain knowledge of precipitation–runoff processes, snowmelt dynamics, and other key hydrological mechanisms into the network architecture and loss function. Although high-quality observations are limited, flood generation involves numerous environmental variables, including meteorological factors such as temperature, humidity, wind speed, and pressure, as well as soil and land-surface characteristics, which can serve as indirect constraints for PINNs. Grid-scale training and validation strategies can further enhance adaptability across heterogeneous terrain, while explicitly incorporating temperature variables allows the models to capture the role of snow and ice melt in modulating runoff, thereby ensuring physical plausibility.

The effectiveness of PINNs for data-fusion hydrological modeling rests on two prerequisites: (1) the integration and sharing of multi-source observational datasets, and (2) regionalized representation of alpine-specific processes. Within this framework, PINNs can achieve reliable simulation and generalization in environments characterized by data scarcity, complex topography, and strong seasonality, thereby providing a robust foundation for improving flood modeling in high-altitude regions.

3) **Toward integrated observational platforms**

Persistent fragmentation of observational datasets remains a critical barrier to progress. Meteorological, hydrological, and radar data are frequently managed by disparate agencies, often using incompatible formats, standards, and access protocols. This institutional and technical disjunction impedes regional data integration, limits the effectiveness of model training, and hinders seamless incorporation into operational systems. At the governance level, we advocate the development of unified metadata and exchange standards (e.g., adoption of widely recognized OGC/INSPIRE protocols [5]) to enable standardized interfaces across agencies. Incentive- and accountability-based sharing mechanisms should also be explored, where data contributions are incorporated into research evaluation and institutional performance metrics to strengthen the willingness to share. On the technical side, an integrated observation platform should not be conceived as centralized storage, but rather as a distributed federated architecture: agencies retain sovereignty over original datasets, while standardized APIs enable interoperability, alleviating concerns about format incompatibility and data security. Within this framework, the platform's core function is to provide real-time cross-source correction and fusion services (e.g., satellite-ground dynamic bias adjustment). Through this dual design of governance and technology, such a platform can enhance both accessibility and interoperability while safeguarding data security and ownership.

Building an integrated observation platform not only helps mitigate the uncertainty arising from sparse networks and biased data but also provides stable support and transferability for emerging approaches such as physics-informed neural networks. The synergy of network optimization, physics-informed modeling, and data governance is essential to enable truly cross-scale flood simulation and the development of the “Four-pre” framework in alpine regions.

## Future perspectives

Flood risk in alpine regions is intricately linked to both societal resilience and ecological integrity, demanding a unified risk governance framework that spans observation, modeling, early warning, and emergency response. Optimized monitoring networks can deliver higher-quality, more representative data under complex terrain and extreme climatic conditions, thereby providing physics-informed neural networks with reliable training samples and physical calibration, and alleviating challenges of data scarcity and model transferability. At the same time, an integrated data-sharing platform ensures that observations from different institutions and sources can be efficiently consolidated, overcoming persistent “data silos” and creating a scalable environment for cross-scale simulations. Observation optimization lays the foundation, physics-informed modeling unlocks potential, and data governance secures systemic integration and long-term sustainability.

Looking ahead, we call for sustained interdisciplinary collaboration to bridge hydrology, atmospheric science, artificial intelligence, and public policy, and to co-develop a comprehensive governance system covering the full cycle from observation and modeling to early warning and emergency response. Only through such a holistic strategy can the field move beyond data-limited forecasting toward an adaptive, intelligence-driven paradigm for flood risk management in alpine regions.

## CRediT authorship contribution statement

**Li Zhou:** Writing – original draft, Investigation, Funding acquisition, Conceptualization. **Haowen Li:** Writing – original draft, Investigation. **Zhongshun Gu:** Resources, Investigation. **Yinan Guo:** Resources, Investigation. **Xiaopeng Wang:** Investigation. **Biqiong Wu:** Investigation, Funding acquisition. **Lingling Wu:** Investigation. **Chun Zhou:** Writing – review & editing, Conceptualization. **Zhipan Niu:** Writing – review & editing, Conceptualization.

## Declaration of competing interest

The authors declare that they have no known competing financial interests or personal relationships that could have appeared to influence the work reported in this paper.
